# Towards an Integrative Approach to Trauma Study

**DOI:** 10.5812/atr.11288

**Published:** 2013-06-01

**Authors:** Abdollah Omidi

**Affiliations:** 1 Trauma Research Center, Kashan University of Medical Sciences, Kashan, IR Iran

**Keywords:** Health, Clinical Psychology, Injury

Recent improvements in psychological, medical, and physiological researches have led to a new way of thinking about health and illness. This conceptualization, which has been known as the bio- psycho-social model, interprets. Recent improvements in psychological, medical, and physiological researches have led to a new way of thinking about health and illness. This conceptualization, which has been known as the bio- psycho-social model, interprets *Health* as the product of a combination of many factors, including biological, psychological, spiritual and social conditions. (1). Health psychology is a branch of psychology, which could be defined as the study of psychological and behavioral effects on health, illness and healthcare (1). It is concerned with understanding how psychological, behavioral and cultural factors, in addition to the biological features which are well understood in medicine, are involved in causing physical health and disease (2). Interactions of the brain, mind, body, and behavior, besides the powerful ways in which emotional, mental, social, spiritual and behavioral factors affect the health are important. Over the past 20 years, mind-body medicine has provided considerable evidences that psychological factors can play a substantial role in the development and progression of diseases (2). it has been reported that the intervention of the mind and body could be effective in the treatment of many somatic and psychiatric problems (3). By training the health care professionals to apply this science in understanding and controlling psychological factors, the level of care will be improved either directly in each patient or indirectly (large-scale) as the public health programs. The concept in which the mind is an integral factor in the healing approaches dating back to more than 2000 years ago. It was also noted by Hippocrates, who recognized the moral and spiritual aspects of healing, and believed that the treatment could occur only with consideration on attitude, environmental influences, and natural remedies (ca. 400 B.C.) (3). While this integrated approach was maintained in traditional healing systems, developments in the Western world by the 16th and 17th centuries led to more and more consideration of physical body as the main or even the only source of disease formation. This separation initiated with the redirection of science, during the Renaissance and Enlightenment eras, with the purpose of enhancing humankind’s control over nature (4). Technological advances such as the invention of microscope, stethoscope, sphygmomanometer and so on, as well as development of refined procedural techniques represented a physical world that was far apart from the world of belief and emotion. The discovery of antibiotics and micro-organisms, and other scientific discoveries have affected the concept of health. Managing and treating a disease became a field of science (i.e., technology) and took precedence over, not a place besides, healing of the soul (5). As medicine separated the mind and the body, scientists of the mind (neurologists) formulated such concepts as the conscious/unconscious, emotional impulses and cognitive delusions, that solidified the perception that diseases of the mind were not “real”, that is, not based on physiology and biochemistry (6). In the 1920s, Walter Cannon demonstrated direct relationship between stress and neuro-endocrine responses in animals. The phrase “fight or flight” applied by Cannon describes the primitive reflexes of sympathetic and adrenal activation in response to perceived danger or any other environmental pressures (e.g. cold, heat). Hans Selye further defined the deleterious effects of stress and distress on health (7). At the same time, technological advances in medicine identified specific pathological changes, and new discoveries in pharmaceuticals, were appearing at a very rapid pace. The disease-based model, the search for a specific pathology and the identification of external cures were paramount, even in psychiatry, however, since the 1960s, mind-body interactions have extensively attracted researchers' attention (7). Studies have demonstrated psychological predictors of health states (8), these variables have roles in anticipation of maintenance and exacerbation of diseases and many disordered behaviors. Trauma is a condition that is related to many variable determinants, so its management actually needs extensive knowledge about psychology, medicine, sociology and cultural issues (9). This implies that confronting with trauma must be based on a team work and multi-disciplinary approach and training courses for experts in this field should be included of a minimum background of various sciences (both humanities and medical sciences). Otherwise, dealing with this aspect of health problems will be exhausting and less yielding, and perhaps poor results in controlling this grave issue in many developing countries is a disintegrated approach (8). I'd suggest that specialized training in trauma studies includes courses on behavior principles, behavioral impairment, personality, psychopathology, motivation and emotions, learning theories, psychology of trauma and socio-cultural issues.
